# A comprehensive consolidation of data on the connection between CDKN2A polymorphisms and the susceptibility to childhood acute lymphoblastic leukemia

**DOI:** 10.1016/j.htct.2024.05.017

**Published:** 2024-10-08

**Authors:** Maryam Aghasipour, Fatemeh Asadian, Seyed Alireza Dastgheib, Abolhasan Alijanpour, Ali Masoudi, Maedeh Barahman, Mohammad Golshan-Tafti, Reza Bahrami, Amirmasoud Shiri, Hossein Aarafi, Kazem Aghili, Hossein Neamatzadeh

**Affiliations:** a College of Medicine, University of Cincinnati, Ohio, USA; b School of Paramedical Science, Shiraz University of Medical Sciences, Shiraz, Iran; c School of Medicine, Shiraz University of Medical Sciences, Shiraz, Iran; d Babol University of Medical Sciences, Babol, Iran; e Shahid Sadoughi University of Medical Sciences, Yazd, Iran; f Firoozgar Hospital, Firoozgar Clinical Research Development Center (FCRDC), Iran University of Medical Sciences, Tehran, Iran; g Islamic Azad University of Yazd, Yazd, Iran; h Neonatal Research Center, Shiraz University of Medical Sciences, Shiraz, Iran; i School of Medicine, Shahid Sadoughi University of Medical Sciences, Yazd, Iran; j Shahid Rahnamoun Hospital, School of Medicine, Shahid Sadoughi University of Medical Sciences, Yazd, Iran; k Mother and Newborn Health Research Center, School of Medicine, Shahid Sadoughi University of Medical Sciences, Yazd, Iran

**Keywords:** Acute lymphoblastic leukemia, Pediatrics, CDKN2A, Polymorphism

## Abstract

**Background:**

Acute lymphoblastic leukemia is the predominant neoplastic ailment in childhood. Prior research has already established noteworthy connections between CDKN2A polymorphisms and susceptibility to this childhood leukemia, however, substantial associations are still awaiting validation. This investigation was undertaken to examine the correlation between CDKN2A polymorphisms and the risk of acute lymphoblastic leukemia in children.

**Methods:**

Acquisition of information encompassed the exploration of diverse databases including PubMed, Scopus, EMBASE, and China National Knowledge Infrastructure (CNKI) until January 10, 2024. An estimation of associations was achieved utilizing odds ratios with 95% confidence intervals.

**Results:**

A total of 22 case-control studies encompassing 10,203 cases of acute lymphoblastic leukemia and 36,424 healthy controls were included. Within this pool of studies, 14 focused on rs3731217, comprising 5396 cases and 15,787 controls, whereas eight studies investigated rs3731249, comprising 4807 cases and 20,637 controls. The aggregated data showed that the rs3731217 variant offers protection against acute lymphoblastic leukemia. Nevertheless, when subgroups are analyzed according to ethnicity, it becomes clear that the rs3731217 polymorphism significantly influences susceptibility, particularly among individuals of Caucasian and African descent with no such association being observed in children of Asian origin. Nevertheless, the rs3731249 polymorphism displayed a noteworthy correlation with vulnerability to pediatric acute lymphoblastic leukemia.

**Conclusion:**

The aggregated data revealed that the rs3731217 variation offers protection against the development of pediatric acute lymphoblastic leukemia and the rs3731249 polymorphism is significantly correlated with susceptibility.

## Introduction

Acute lymphoblastic leukemia (ALL [MIM: 613,065]) is the predominant malignant condition affecting children, posing a significant threat to their well-being and imposing substantial economic and psychological burdens on society and their families. ALL accounts for about 75% of all leukemias with B-cell ALL (B-ALL) being the main type in children, with a peak incidence at the ages of 2–5 years old.^1^, ^2^, ^3^ While developed countries experience higher incidence rates, disparities exist within and between countries due to various genetic, environmental, and socioeconomic factors.^4^ Gender differences also exist, with boys being slightly more prone to developing ALL.^5^ This leukemia is a clonal disease of the hematopoietic system, evolving from immature B or T cells; it is the most common cancer diagnosed in under 15-year-old children.^6^^,^^7^ ALL is a heterogeneous hematologic malignancy in the context of genetic background, clinical manifestation and prognosis. The incidence and prognosis of childhood ALL varies by age, gender, and ethnicity.^8^, ^9^, ^10^ According to the World Cancer Research Fund, the estimated global incidence of ALL in developed countries ranges from 1.5 to 4 cases per 100,000 people annually. Factors like environmental influences, genetic susceptibility, and healthcare accessibility play a role in these elevated rates.^11^ The Global Burden of Disease online database indicates that the number of newly diagnosed leukemia cases rose from 354,500 in 1990 to 518,500 in 2017.^8^ The number of ALL cases increased by 30.81% globally from 1990 to 2017, indicating that ALL might become more important as a public health concern worldwide.^2^^,^^12^^,^^13^ For the past five decades, scholars in this field have dedicated themselves to employing innovative technologies to continuously explore novel molecular biological targets, leveraging them for precise disease prognosis, classification, and the development of new pharmaceuticals. However, the exact mechanisms underlying ALL remain incompletely understood. Multiple factors are believed to contribute to the onset of ALL, including maternal alcohol consumption during pregnancy, maternal history of prior miscarriages, advanced paternal age, low birth weight, exposure to ionizing radiation, infectious agents, pesticides, and genetic predisposition.^14^, ^15^, ^16^, ^17^, ^18^

Genome-wide association studies (GWAS) have discovered several risk loci for susceptibility to pediatric ALL accounting for 21% of the heritability.^19^ Notably, the missense variant rs3731249 of chromosome 9p21.3 in the CDKN2A gene is linked to a three-fold higher risk of ALL in European and Hispanic children.^20^ Furthermore, epigenetic mechanisms such as DNA methylation have been implicated in mediating the effects of genetic risk loci for childhood ALL, with specific CpG sites influencing the risk associated to single nucleotide polymorphisms (SNPs) in genes like IKZF1 and ARID5B.^20^ Combined analyses of GWAS datasets have also highlighted gene associations including ARID5B, IKZF1, CDKN2A/2B, and PIP4K2A, providing insight into the intricate genomic architecture that influences susceptibility to ALL.^21^ The 9p21.3 region encompasses three notable tumor suppressor genes, namely CDKN2A, CDKN2B, and MTAP, as well as a long noncoding RNA called ANRIL (antisense noncoding RNA at the ink4 locus non-coding gene).^22^ CDKN2A is one of the major genomic hotspots for disease heritability and susceptibility locus identified to date.^23^ The gene products of CDKN2A act as tumor suppressors, playing a direct role in the regulation of the cell cycle and the negative control of cell proliferation.^22^^,^^24^ The CDKN2A gene is composed of two introns and three exons and generates various transcripts through alternative splicing, resulting in the production of at least three distinct proteins, including p16Ink4a and p14ARF. These two proteins collaborate to regulate CDK4 and p53, thereby directly influencing the negative regulation of cell transition from the G1 phase to the S phase.^25^ Deletion or mutation of the CDKN2A gene leads to uncontrolled CDK4 activity, which in turn induces malignant cell proliferation.^26^ Simultaneously, CDKN2A is recognized as a key gene in the process of cellular senescence and contributes to the aging of the human body.^27^ Repression of the CDKN2A locus is observed in most normal tissues and serves as a promising molecular biomarker for various human aging phenotypes, including atherosclerotic disease, metabolic diseases, glaucoma, and malignancies.^23^^,^^28^^,^^29^ Treatment alternatives for cases of leukemia with CDKN2A mutations encompass the targeting of the CDK2-SKP2 axis, the utilization of the small-molecule CDK4/6 inhibitor known as palbociclib, and the application of the innovative CDK2/9 inhibitor referred to as fadraciclib.^30^ A Phase 1 clinical trial investigating the combination of palbociclib with standard chemotherapy has provided complete responses in both pediatric and young adult patients afflicted with relapsed/refractory B-ALL and T-cell ALL (T-ALL), as well as lymphoma.^31^

High-throughput sequencing technologies showed that the sequence of the CDKN2A gene is considerably polymorphic.^32^ Both clinical and experimental investigations have revealed that the genomic locus of CDKN2A is linked to susceptibility of childhood ALL in various ethnic populations.^32^, ^33^, ^34^ While genetic alterations at this locus are well-established contributors to tumorigenesis, there is also evidence suggesting that specific disease-associated polymorphisms may modulate the risk of developing ALL in children.^33^ Furthermore, the majority of these variants consistently display the same directional effects across different ethnic groups, with limited heterogeneity observed.^35^ In order to shed further light on this matter, the objective of this study was to assess the correlation between widely studied polymorphisms in the CDKN2A gene and the risk of pediatric ALL through a comprehensive meta-analysis including all relevant case-control studies.

## Materials and methods

### Search approach

The acquisition of ethical approval was deemed unnecessary for the present investigation, given its nature as a systematic review and meta-analysis. Furthermore, this scholarly inquiry strictly adhered to the guidelines set forth by PRISMA (Preferred Reporting Items for Systematic Reviews and Meta-Analyses).

The search for case-control studies investigating the relationship between CDKN2A polymorphisms and the risk of pediatric ALL was conducted using various online databases, including PubMed, Web of Science, Europe PMC, ResearchGate, Elsevier, Cochrane Library, EMBASE, SciELO, Grey Literature, WanFang, VIP Information Consulting Company (VIP), CENTRAL, Proteomics, Google Scholar, Wanfang Data Company, Chaoxing, Chinese Medical Citation Index (CMCI), Sinomed, Baidu, Chinese Medical Current Contents (CMCC), Chinese Biomedical Database (CBD), Chinese National Knowledge Infrastructure (CNKI) and Weipu Periodical Database. This study included publications until January 10, 2024. To refine the search, specific keywords and terms were employed, such as ‘childhood’ or ‘acute lymphoblastic leukemia’ in combination with ‘9p21.3′ or ‘cyclin-dependent kinase inhibitor 2A’ or ‘CDKN2A,’ and ‘Gene’ or ‘Genetic’ or ‘DNA Sequence’ or ‘single-nucleotide polymorphism’ or ‘SNPs’ or ‘polymorphism’ or ‘genotype’ or ‘frequency’ or ‘mutation’ or ‘mutant’ or ‘allele’ or ‘variation’ or ‘variant.’ Additionally, a manual search of references in relevant articles and reviews was conducted to identify additional pertinent literature. The scope of the search was limited to human studies without any language restrictions. Furthermore, the reference lists of applicable reviews and eligible publications were reviewed to identify other potential sources of information.

### Inclusion and exclusion criteria

All the studies that were included in this analysis fulfilled specified criteria. These criteria included: 1) the studies needed to have a case-control or cohort design, and needed to be reported in English, Russian, or Chinese; 2) the studies had to investigate the correlation between CDKN2A polymorphisms and the risk of pediatric ALL; 3) the case group consisted of children with ALL, while the control group consisted of healthy children; and 4) the studies needed to have sufficient and accessible data in order to calculate the odds ratios (ORs) and 95% confidence intervals (95% CIs). On the other hand, certain exclusion criteria were applied. These criteria included: 1) case reports, case series, letters, commentaries, editorials, reviews, animal experiments, in vitro cell experiments, conference papers, and meta-analyses were not considered; 2) studies that had incomplete literature data or where the text could not be obtained after contacting the author were excluded; 3) studies that presented inadequate or unapproachable data were not included; 4) studies that were conducted on family members were also excluded; and 5) data that were duplicated or overlapped with other studies were not considered.

### Data extraction

According to the aforementioned inclusion and exclusion criteria, two researchers independently examined the references, obtained data, and cross-verified the findings. Any discrepancies were resolved through deliberation or a meeting involving a third scientist. During the review of the literature, the initial assessment involved reading the title and abstract to eliminate irrelevant material, followed by a thorough examination of the complete text to determine its eventual inclusion. The literature that adhered to the set standards yielded the following key information: the primary author's name, the year of publication, the country of origin, the ethnicity, the genotyping techniques employed, the total number of cases and controls, the frequencies of genotypes for CDKN2A polymorphisms in cases and healthy controls, the evaluation of Hardy-Weinberg equilibrium (HWE), as well as the minor allele frequencies (MAFs) in children of good health status, all necessary pieces of information. In instances where the same investigator(s) reported multiple studies using duplicated or overlapping records, only the most recently published data or the study with the largest sample size was considered for inclusion.

### Statistical analysis

The HWE was determined using the chi-Square (χ^2^) test within a healthy population encompassed in a solitary investigation, employing readily accessible online software.^36^ A p-value below the threshold of 0.05 was regarded as possessing statistical significance. The association between CDKN2A polymorphisms and the risk of ALL was assessed using ORs with 95% CIs. The statistical significance of the combined data was determined by applying the Z-test to the difference between the population mean and the sample mean. This meta-analysis took into account five genetic models, which encompassed allelic (B versus A), homozygote (BB versus AA), heterozygote (BA versus AA), dominant (BB+BA versus AA), and recessive (BB versus BA+AA) models. The chi-square test is commonly used to assess the significance of heterogeneity, with a significance level of p-value <0.05. Furthermore, according to Cochrane, the level of heterogeneity between studies was defined on a scale of 0–100%.^37^ If I^2^ was greater than 50%, random-effect models (DerSimonian-Laird method) were used. Otherwise, fixed-effect models (Mantel-Haenszel method) were employed for interpretation. Sensitivity analysis was conducted by systematically omitting one study at a time to assess the robustness of the results. Publication bias was assessed using Begg's test, where the standard error of each study was plotted against its OR, and Egger's test visually examined the funnel plot for asymmetry. If publication bias was present, the trim-and-fill method was used to adjust the conclusions accordingly. Data synthesis from primary studies was conducted using the Comprehensive meta-analysis (Version 4.0) software (Biostat, USA). For statistical significance, a two-sided p-value <0.05 was deemed significant.

### Quality score appraisal

The Newcastle-Ottawa Score (NOS) was implemented in order to evaluate the excellence and caliber of the chosen individual studies, taking into account the diverse facets of the methodology employed for observational research. This benchmark was employed to evaluate three distinct components, namely the selection of cases, the comparability of groups, and the determination of exposure, and was further comprised of eight different items. Within the selection and exposure categories, a research item of high quality would be awarded a solitary star, whereas a category that demonstrated comparability could potentially receive a maximum of two stars. The values assigned during the quality assessment process spanned from zero stars, indicative of the poorest quality, to a maximum of nine stars, representing the highest caliber. Consequently, studies that attained a score equal to or greater than seven were classified as being of high quality. In a general sense, any study that achieved a minimum score of five points was deemed suitable for inclusion in the meta-analysis, and any differences or disagreements that arose were effectively resolved through discussion and consensus.

## Results

### Study characteristics

**Figure 1:**
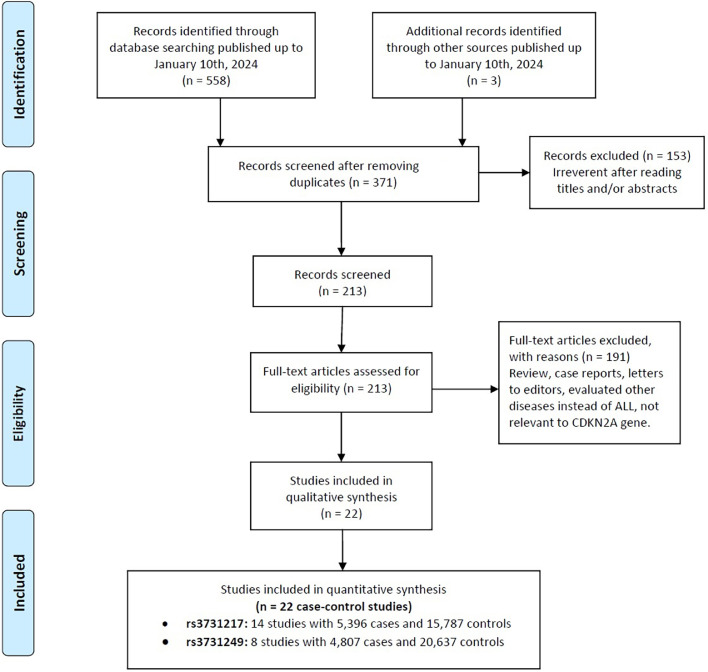
Flow diagram of the study selection process.

The flowchart illustrating the comprehensive selection process is shown in Figure 1. Initially, an extensive search was conducted across multiple electronic databases, as well as a meticulous manual examination of the entire contents page by page, resulting in the identification of a total of 561 papers. Upon careful scrutiny of the research title and abstract, 190 duplicate documents and 158 irrelevant articles were excluded, leaving 213 articles that were thoroughly perused in their entirety. Ultimately, 14 independent publications ^22^^,^^32^^,^^38^, ^39^, ^40^, ^41^, ^42^, ^43^, ^44^, ^45^, ^46^, ^47^, ^48^, ^49^ with 22 case-control studies that adhered to our criteria were included, encompassing 10,203 patients afflicted with ALL and 36,424 healthy controls. Within this pool of studies, 14 focused on rs3731217, comprising 5396 cases and 15,787 controls, whereas eight studies investigated rs3731249, comprising 4807 cases and 20,637 controls. These studies were published between 2010 and 2023. It is noteworthy that all of the studies included in this analysis were written in English. Several genotyping techniques, namely polymerase chain reaction (PCR), kompetitive allele specific PCR (KASP), array based imputation, illumina array, fluidigm dynamic array, high-resolution melting (HRM), PCR-restriction fragment length polymorphism (PCR-RFLP), and allele-specific polymerase chain reaction (AS-PCR), were employed to determine the genotypes of the polymorphisms. These studies were conducted in various countries including the United Kingdom, Poland, France, Germany, the United States, Latvia, Yemen, Iran, Palestine, Canada, and Spain. Of these studies, 14 focused on individuals of Caucasian descent, while five specifically targeted Asian children. Additionally, two studies concentrated on African children, and one study examined Hispanic children. The NOS obtained from the included studies reached a value greater than 7, as indicated in Table 1. Therefore, it can be inferred that the overall quality of the included studies was of a high standard. It is of significant importance to acknowledge that the occurrence rate of the rs3731217 and rs3731249 polymorphisms in the control group exactly matched the expected frequencies according to the HWE (p-value >0.05).

**Table 1:** Characteristics of the studies included in the meta-analysis.

First Author/Year	Ethnicity (Country)	Genotyping Methods	Case/Control	Patients					Healthy Control					MAFs	HWE	NOS
				Genotypes			Alleles		Genotypes			Alleles				
rs3731217				TT	TC	CC	T	C	TT	TC	CC	T	C			
Vijayakrishnan 2010	UK(Asian)	KASP	190/182	148	40	2	336	44	138	43	1	319	45	0.124	0.222	7
Pastorczak 2011	Poland(Caucasian)	KASP	387/715	304	76	7	684	90	551	148	16	1250	180	0.126	0.112	9
Orsi 2012	France(Caucasian)	Illumina array	441/1984	338	96	7	772	110	1452	486	46	3390	578	0.146	0.481	10
Burmeister 2014	Germany(Caucasian)	TaqMan	322/1503	260	60	2	580	64	1092	379	32	2563	443	0.147	0.895	8
Vijayakrishnan 2015	UK(Caucasian)	Illumina array	824/5200	659	149	16	1467	181	3778	1311	111	8867	1533	0.147	0.826	9
Vijayakrishnan 2015	UK(Caucasian)	Illumina array	834/2024	685	140	9	1510	158	1523	468	33	3514	534	0.132	0.666	9
Hungate 2016	USA(Caucasian)	ABI	1406/1384	1166	224	16	2556	256	1052	313	19	2417	351	0.127	0.429	9
Hungate 2016	USA(African)	ABI	203/1243	172	31	0	375	31	1044	187	12	2275	211	0.085	0.266	9
Hungate 2016	USA(Hispanic)	ABI	391/978	344	46	1	734	48	810	157	11	1777	179	0.092	0.279	9
Kreile 2016	Latvia(Caucasian)	PCR-RFLP	76/121	64	12	0	140	12	95	26	0	216	26	0.107	0.107	8
Gharbi 2016	Tunis(African)	PCR	58/150	13	31	14	57	59	21	78	51	120	180	0.600	0.307	9
Al-Absi 2017	Yemen(Asian)	Dynamic Array	136/153	104	29	3	237	35	111	36	6	258	48	0.157	0.171	9
Bardsiri 2022	Iran(Asian)	HRM	50/50	48	2	0	98	2	49	1	0	99	1	0.010	0.943	7
Al-Zaya 2023	Gaza(Asian)	AS-PCR	78/100	12	62	4	86	70	24	45	31	93	107	0.535	0.339	7
rs3731249				**CC**	**CT**	**TT**	**C**	**T**	**CC**	**CT**	**TT**	**C**	**T**			
Healy 2007	Canada(Caucasian)	PCR	227/275	195	30	2	420	34	256	18	1	530	20	0.036	0.273	9
Vijayakrishnan 2010	UK(Caucasian)	Illumina array	835/2024	722	101	12	1545	125	1916	106	2	3938	110	0.027	0.670	9
Vijayakrishnan 2010	UK(Caucasian)	Illumina array	823/5198	750	69	4	1569	77	4933	261	4	10,127	269	0.026	0.774	9
Vijayakrishnan 2010	UK(Caucasian)	KASP	519/1016	472	45	2	989	49	974	41	1	1989	43	0.021	0.409	9
Xu 2015	USA(Caucasian)	Illumina array	1773/10,241	1541	224	8	3306	240	9615	619	7	19,849	633	0.031	0.358	8
Xu 2015	USA(Caucasian)	Illumina array	409/1599	357	45	7	759	59	1467	130	2	3064	134	0.042	0.615	8
Gutierrez-Camino 2017	Spain(Caucasian)	PCR	171/234	142	28	1	312	30	217	16	1	450	18	0.038	0.247	9
Bardsiri 2022	Iran(Asian)	HRM	50/50	41	8	1	90	10	49	1	0	99	1	0.010	0.943	7

KASP: Kompetitive allele specific polymerase chain reaction; HRM: High-resolution melting; PCR-RFLP: Polymerase chain reaction-restriction fragment length polymorphism; AS-PCR: Allele-specific polymerase chain reaction; MAF: Minor allele frequency; HWE: Hardy-Weinberg equilibrium; NOS: Newcastle-Ottawa score.

### Quantitative data synthesis

#### CDKN2A rs3731217 *polymorphism*

**Figure 2:**
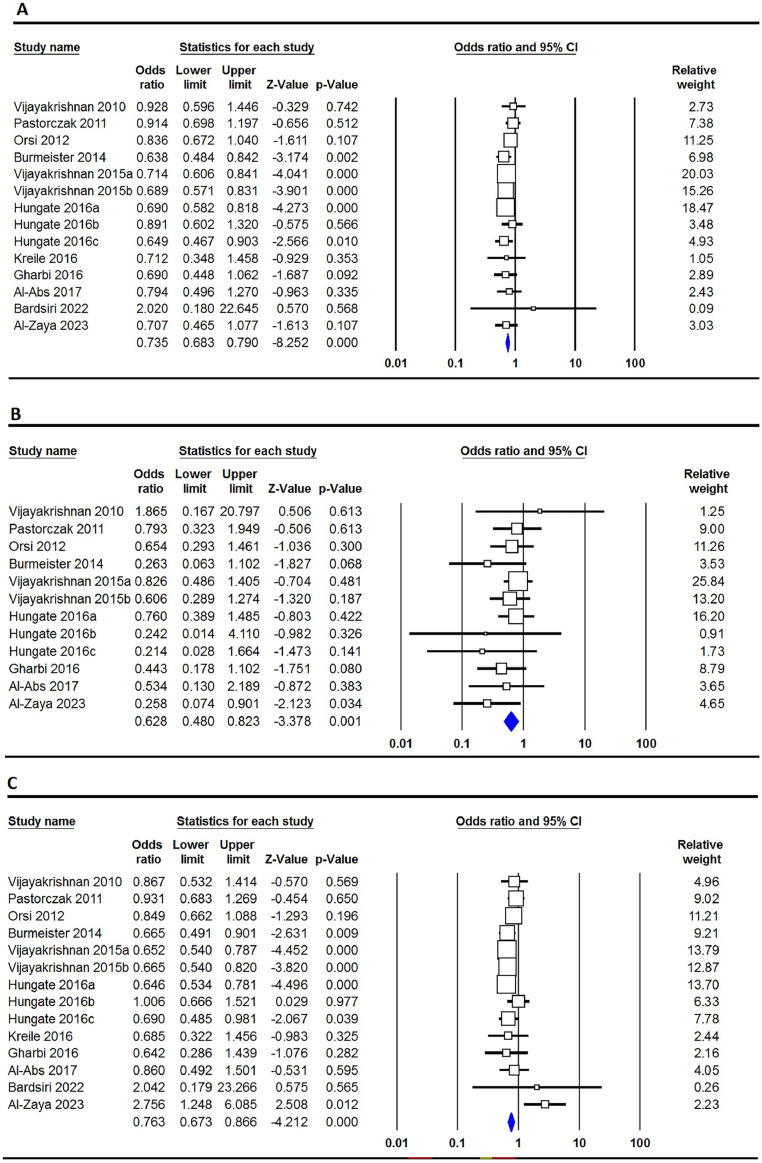

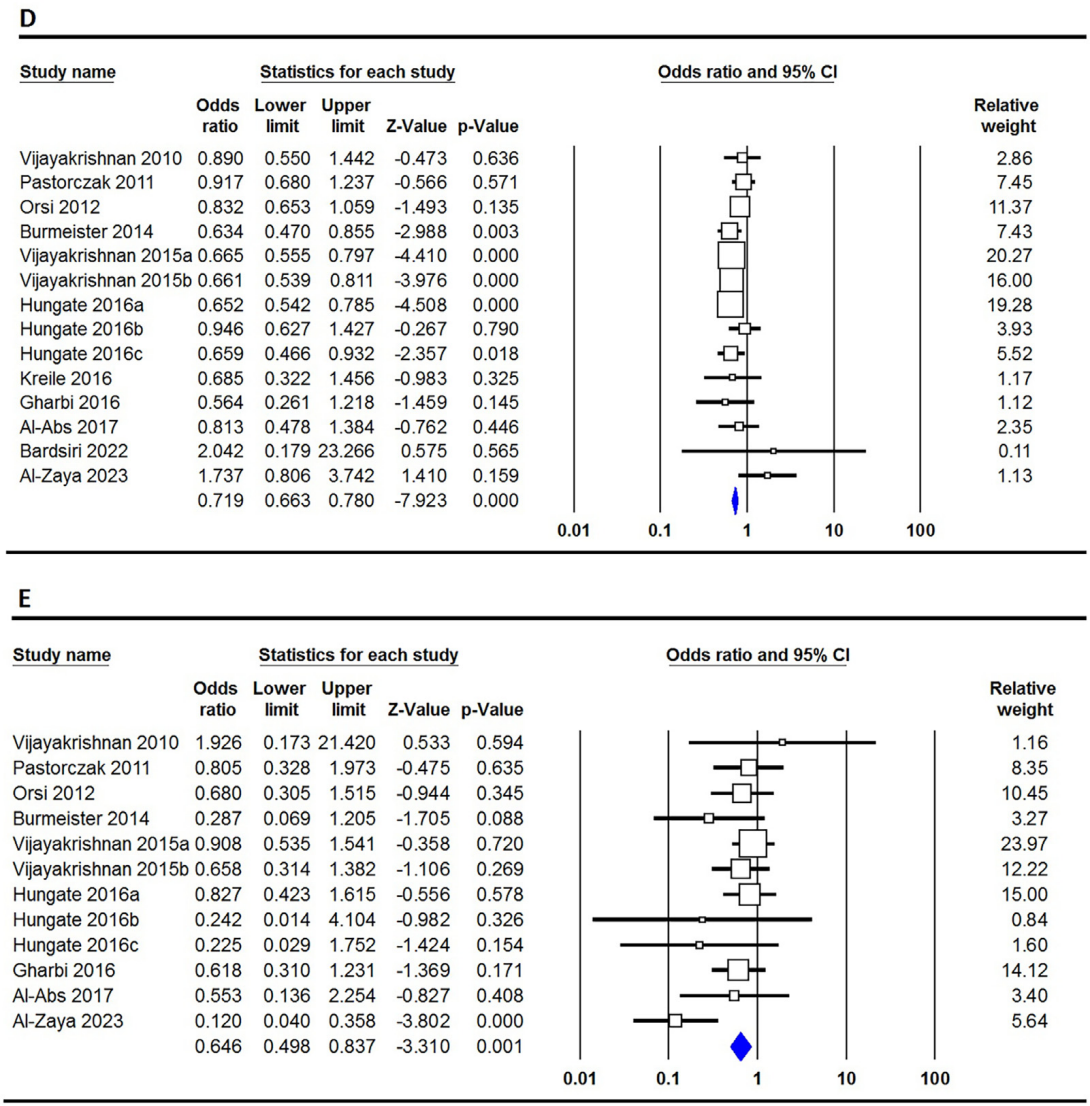
Forest plots for the association of CDKN2A rs3731217 polymorphism and risk of pediatric acute lymphoblastic leukemia in overall population using different models - A: allele (C versus T); B: homozygote (CC versus TT); C: heterozygote (CT versus TT); D: dominant (CC+CT versus TT); and E: recessive (CC versus CT+TT).

The correlation between the rs3731217 polymorphism of **CDKN2A** and the risk of ALL is concisely summarized in Table 2. Our extensive analysis of combined data undeniably demonstrates that the rs3731217 polymorphism plays a protective role in the development of ALL in the overall population. This assertion is strengthened by the examination of all five genetic models, namely the allele model (C versus T: OR = 0.735; 95% CI: 0.683–0.790; p-value ≤0.001 - Figure 2A), the homozygote model (CC versus TT: OR = 0.628; 95% CI: 0.480–0.823; p-value = 0.001 - Figure 2B), the heterozygote model (CT versus TT: OR = 1.359; 95%: CI 0.673–0.866; p-value ≤0.001 - Figure 2C), the dominant model (CC+CT versus TT: OR = 0.719; 95% CI: 0.663–0.780; p-value ≤0.001 - Figure 2D), and the recessive model (CC versus CT+TT: OR = 0.646; 95% CI: 0.498–0.837; p-value = 0.003 - Figure 2E). However, upon conducting subgroup analysis stratified by ethnicity, it becomes evident that the rs3731217 polymorphism has a noticeable impact on the risk of ALL specifically within the Caucasian population. This is demonstrated by the consistent findings across four genetic models, namely the allele model (C versus T: OR = 1.233; 95% CI: 1.043–1.450; p-value = 0.011), the homozygote model (CC versus TT: OR = 1.540; 95% CI: 1.117–2.122; p-value = 0.008), the dominant model (CC+CT versus TT: OR = 1.206; 95% CI: 1.002–1.453; p-value = 0.048), and the recessive model (CC versus CT+TT: OR = 1.522; 95% CI: 1.107–2.094; p-value = 0.010). Furthermore, it is observed that in African children, under two genetic models, the homozygote model (CC versus TT: OR = 1.670; 95% CI: 1.228–2.270; p-value = 0.008) and the dominant model (CC+CT versus TT: OR = 1.443; 95% CI: 1.103–1.888; p-value = 0.007), similar trends are observed. However, no such impact is seen in Asian children.

**Table 2:** Summary of meta-analysis for association of CDK2N1 genetic variants with risk of ALL.

Polymorphism	Genetic Model	Type of Model	Heterogeneity		Odds Ratio				Publication Bias	
			I^2^ (%)	P_H_	OR	95% CI	Z_test_	P_OR_	P_Beggs_	P_Eggers_
rs3731217										
Overall	C vs. T	Fixed	0.00	0.743	0.735	0.683–0.790	−8.252	≤0.001	0.273	0.202
	CC vs. TT	Fixed	0.00	0.725	0.628	0.480–0.823	−3.378	0.001	0.114	0.060
	CT vs. TT	Random	42.76	0.045	0.763	0.673–0.866	−4.212	≤0.001	0.062	0.028
	CC+CT vs. TT	Fixed	19.39	0.242	0.719	0.663–0.780	−7.923	≤0.001	0.125	0.057
	CC vs. CT+TT	Fixed	26.75	0.182	0.646	0.498–0.837	−3.000	0.003	0.149	0.129
Ethnicity										
Asian	C vs. T	Random	88.08	≤0.001	1.178	0.887–1.566	1.130	0.258	0.234	0.463
	CC vs. TT	Fixed	77.64	≤0.001	1.193	0.642–2.217	0.559	0.576	1.000	0.537
	CT vs. TT	Random	85.61	≤0.001	1.098	0.791–1.523	0.557	0.577	0.137	0.238
	CC+CT vs. TT	Random	87.42	≤0.001	1.162	0.840–1.608	0.906	0.365	0.092	0.354
	CC vs. CT+TT	Random	72.62	≤0.001	1.124	0.678–1.864	0.454	0.650	0.964	0.491
Caucasian	C vs. T	Random	45.73	0.048	1.233	1.043–1.450	2.530	0.011	1.000	0.462
	CC vs. TT	Fixed	16.47	0.287	1.540	1.117–2.122	2.637	0.008	0.876	0.550
	CT vs. TT	Random	48.49	0.035	1.144	0.937–1.397	1.324	0.186	0.640	0.907
	CC+CT vs. TT	Random	46.11	0.046	1.206	1.002–1.453	1.977	0.048	1.000	0.661
	CC vs. CT+TT	Fixed	16.88	0.283	1.522	1.107–2.094	2.585	0.010	0.755	0.586
African	C vs. T	Fixed	0.00	0.764	1.245	1.005–1.542	2.008	0.045	1.000	0.802
	CC vs. TT	Fixed	0.00	0.537	1.156	0.757–1.766	0.672	0.502	1.000	0.289
	CT vs. TT	Fixed	24.82	0.264	1.670	1.228–2.270	3.268	0.001	1.000	0.564
	CC+CT vs. TT	Fixed	0.00	0.615	1.443	1.103–1.888	2.679	0.007	1.000	0.766
	CC vs. CT+TT	Fixed	0.00	0.714	0.947	0.635–1.411	−0.269	0.788	1.000	0.185
rs3731249										
Overall	T vs. C	Fixed	31.62	0.175	2.235	2.016–2.478	15.275	≤0.001	0.536	0.591
	TT vs. CC	Fixed	0.00	0.770	7.293	4.057–13.111	6.640	≤0.001	0.107	0.141
	TC vs. CC	Fixed	38.14	0.125	2.124	1.902–2.373	13.351	≤0.001	0.536	0.701
	TT+TC vs. CC	Fixed	34.25	0.155	2.219	1.991–2.473	14.427	≤0.001	0.710	0.631
	TT vs. TC+CC	Fixed	0.00	0.752	6.835	3.802–12.285	6.424	≤0.001	0.063	0.135

#### CDKN2A rs3731249 *polymorphism*

**Figure 3:**
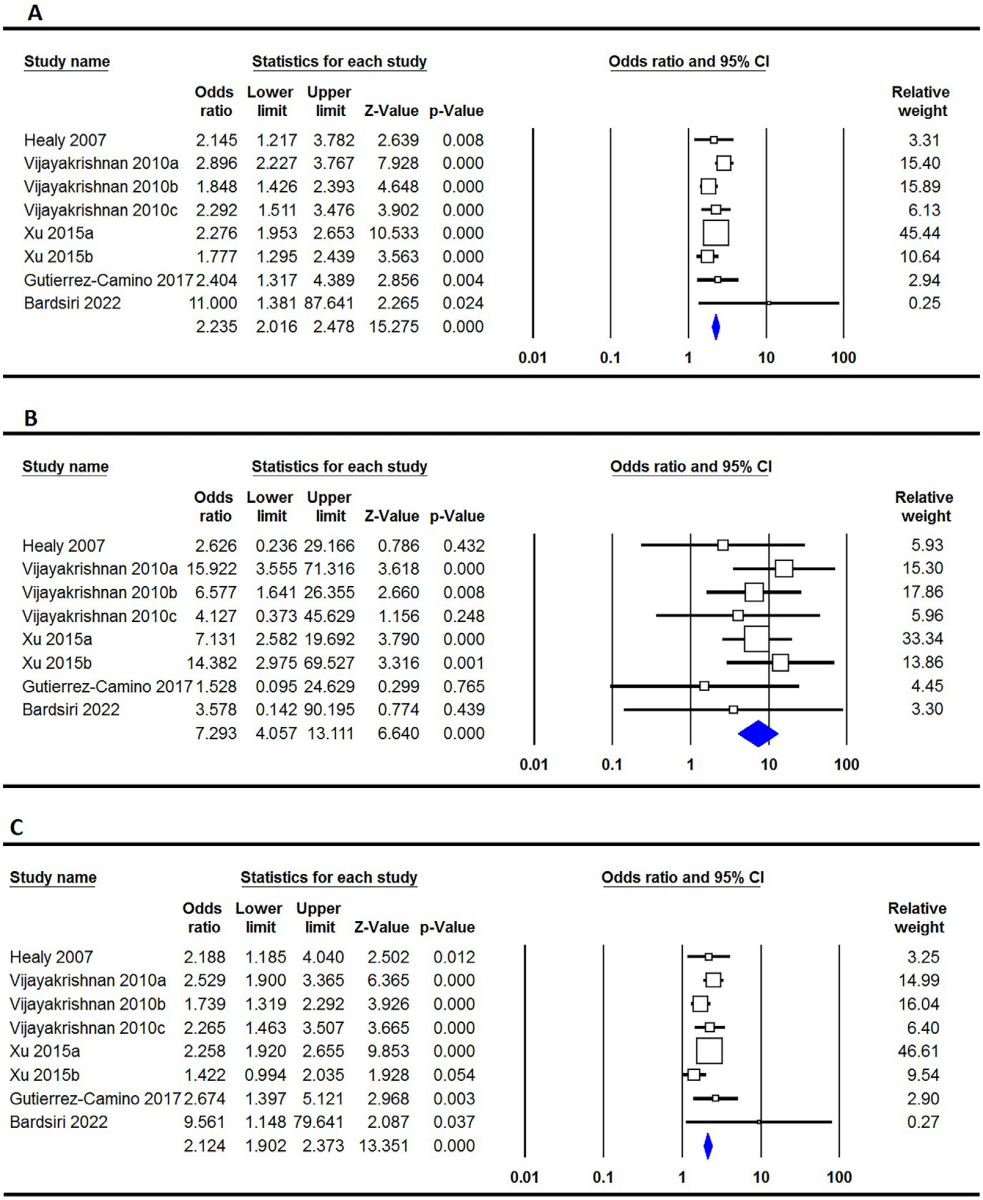

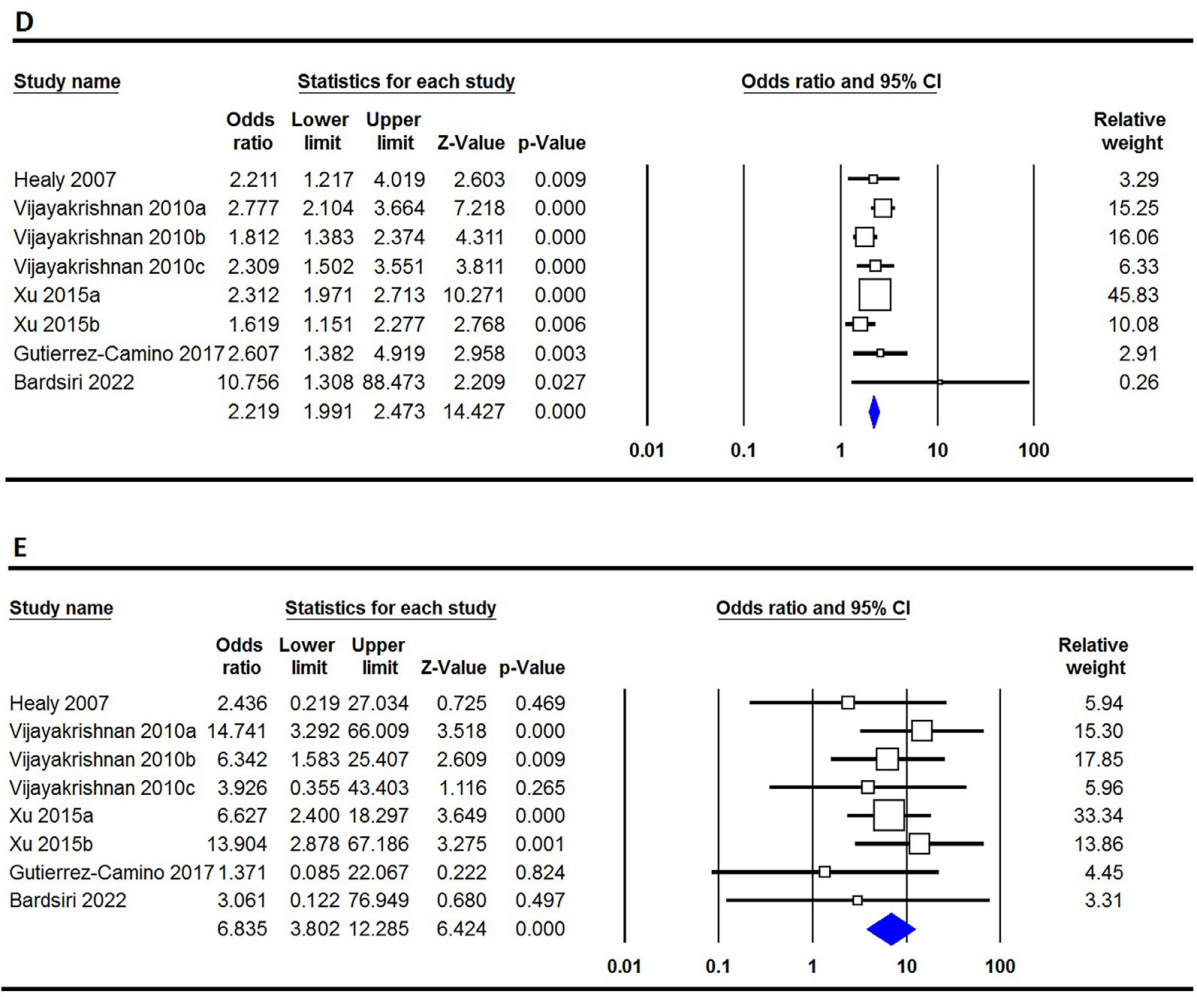
Forest plots for the association of CDKN2A rs3731249 polymorphism and risk of pediatric acute lymphoblastic leukemia in overall population using different models - A: allele (T versus C); B: homozygote (TT versus CC); C: heterozygote (TC versus CC); D: dominant (TT+TC versus CC); and E: recessive (TT versus TC+CC).

The presentation of the relationship between the rs3731249 genetic variation in the **CDKN2A** gene and the susceptibility to ALL is outlined in Table 2. The pooled data indicates that the rs3731249 polymorphism is significantly associated with the risk of ALL on a global scale, according to all five genetic models: the allele model (T versus C: OR = 2.235; 95% CI: 2.016–2.478; p-value ≤0.001 - Figure 3A), the homozygote model (TT versus CC: OR = 7.293; 95% CI: 4.057–13.111; p-value ≤0.001 - Figure 3B), the heterozygote model (TC versus CC: OR = 2.124; 95% CI: 1.902–2.373; p-value ≤0.001 - Figure 3C), the dominant model (TT+TC versus CC: OR = 2.219; 95% CI: 1.991–2.473; p-value ≤0.001 - Figure 3D), and the recessive model (TT versus TC+CC: OR = 6.835; 95% CI: 3.802–12.285; p-value ≤0.001 - Figure 3E).

#### Sensitivity analysis and between-study heterogeneity tests

A sensitivity analysis was conducted whereby one individual study was removed at a time to examine the impact of each individual study on the combined data. Overall, no discernible changes in the results were observed, suggesting that our pooled data is statistically robust and stable. Furthermore, we performed a sensitivity analysis by excluding studies that violated the HWE, and the results remained unchanged.Table 2 demonstrates significant heterogeneity between studies for both **CDKN2A** rs3731217 and rs3731249 polymorphisms across all five genetic models. Consequently, a stratified analysis was carried out based on ethnicity, source of controls, and genotyping methods in order to identify the source of heterogeneity. Among the stratified analyses, a significant reduction in heterogeneity was observed among Caucasian participants, suggesting that ethnicity may contribute to the substantial heterogeneity observed in our pooled data.

#### Publication bias

**Figure 4:**
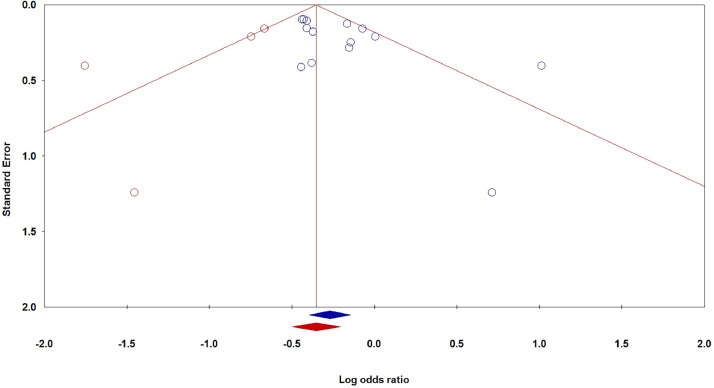
Begg's funnel plot of publication bias test for correlation between the CDKN2A rs3731217 polymorphism and risk of pediatric ALL in overall population under the heterozygote model (CT versus TT).

The Begg's funnel plot and Egger's test are utilized in order to scrutinize potential bias that may exist in the current body of literature. No indications of asymmetry were observed on visual examination of the funnel plot. Furthermore, the results of Egger's test indicated no significant bias in publications across all five genetic models for studies pertaining to the rs3731249 polymorphism. Nonetheless, an analysis of the publication bias did reveal significant bias for studies on the rs3731217 polymorphism under the heterozygote model (P_Beggs_ = 0.062 and P_Eggers_ = 0.028 - Figure 4). As a result, the Duval and Tweedie non-parametric 'trim and fill' method was employed to account for the publication bias. The meta-analysis conducted with and without the 'trim and fill' adjustment yielded consistent results, thereby demonstrating the statistical robustness of the current meta-analysis.

## Discussion

ALL is the predominant neoplasm among the pediatric population ^50^ with modifications in the CDKN2A gene having been postulated to have a significant influence on the development of this malignancy. Two specific polymorphisms located at the CDKN2A locus have been identified as having an impact on susceptibility to ALL as determined through comprehensive GWAS. Additionally, the presence of the CT genotype and increased frequency of the T allele in the CDKN2A SNP rs3731249 are significantly associated with an elevated risk of developing ALL.^22^^,^^51^ Furthermore, GWAS have identified numerous genetic variations linked to a higher susceptibility to ALL with these genetic variations being located within key genes such as IKZF1, ARID5B, and PIP4K2A. The influence of these genetic risk loci on the risk of developing ALL may be mediated through epigenetic mechanisms, specifically DNA methylation. An example of this is the role of DNA methylation at CpG cg01139861 in the promoter region of IKZF1, which mediates the effects of the IKZF1 risk polymorphism rs78396808.^21^^,^^39^ These findings were subsequently corroborated by numerous replication studies, specifically targeting the aforementioned genetic variants.

The minor G allele, known as rs3731217, has been linked to an increased expression of exon 3. This particular exon covers the 3′-UTR region of the CDKN2A gene, leading to elevated levels of the CDKN2A tumor suppressor protein. 52 This meta-analysis, based on a total of 14 studies focused on rs3731217 comprising 5396 cases and 15,787 controls, showed that the rs3731217 variant has a protective role in development of pediatric ALL. It has been previously documented that the rs3731217 polymorphism is associated with ALL in individuals of Caucasian descent. Furthermore, it has been observed that the minor G allele within this polymorphism provides a protective effect against pediatric B-cell precursor ALL. 53 In contrast, the prevalence of the T allele has been found to be similar in both pediatric ALL cases and controls within a Tunisian population. 49 In 2018, Zhou et al. conducted a meta-analysis to reassess the correlation between the two polymorphisms known as rs3731217 and rs3731249, and susceptibility to ALL. The investigation encompassed a total of 7922 cases and 21,503 controls for rs3731217, revealing a statistically significant association between this polymorphism and the risk of ALL. However, this correlation was found to be contingent upon race, with the polymorphism being predominantly linked to ALL susceptibility in individuals of Caucasian lineage. The researchers made the discovery that the presence of the C allele showed a statistically significant 0.72-fold increase in risk for developing ALL in comparison to the T allele. The outcome of their research findings unequivocally diverged from the conclusions derived from our own investigation on rs3731217, displaying a clear and discernible disparity in the data and outcomes obtained. 33 In 2015, Walsh et al. employed SNP genotyping and imputation-based fine-mapping techniques to examine a multiethnic ALL population consisting of 1464 cases and 3279 controls. Their objective was to identify variants with significant impact within 9p21.3. They successfully identified the CDKN2A rs3731249 polymorphism, which exhibited a 2% allele frequency in controls and was found to confer a three-fold increased risk of ALL in children of European ancestry as well as Hispanic children. Furthermore, it has been noted that among the group of 17 individuals who exhibited allelic imbalance at CDKN2A in their tumors, 14 individuals demonstrated a preference for retaining the allele associated with increased risk, while simultaneously losing the allele associated with protection. This observation implies that the risk allele confers a distinct advantage in promoting tumor growth.54

Due to the location of the rs3731249 genetic variant within the coding region of the gene, there was an ensuing alteration from alanine to threonine at position 148 (A148T).^33^ Consequently, allelic imbalanced was assessed in somatic leukemia cells obtained from individuals possessing a heterozygous genotype of rs3731249 in two separate studies involving 15 and 35 cases. The findings revealed that the variant allele was preferentially retained to a significant extent through either copy number variation or post-transcriptional inactivation during the development of leukemia. In their study, Zhou et al. conducted an evaluation on the correlation between the genetic variant rs3731249 and susceptibility to childhood ALL. The research team incorporated a comprehensive analysis of five articles, aggregating data from a substantial cohort of 6295 cases and 24,181 controls. The findings from their investigation revealed a significant association between the minor allele (T) and an increased risk of ALL. This association was supported by statistical evidence, with a *p*-value <0.00001, an OR of 2.26, and a 95% CI ranging from 2.06 to 2.48.^33^ Zhang et al. reported that the deletions of CDKN2A/B were associated with unfavorable prognosis in both adult and pediatric patients with ALL thereby suggesting that the assessment of CDKN2A/B status could potentially enhance risk stratification of ALL patients. Furthermore, the CDKN2A missense variant (rs3731249) was found to confer a 3-fold heightened risk of developing ALL in children of European descent as well as Hispanic children.^52^ In 2017, Dong et al. undertook a meta-analysis with the intention of providing a more lucid comprehension of the correlation between CDKN2A polymorphisms and the risk of cancer. The results of the meta-analysis indicated that the CDKN2A rs3731249, rs11515, and rs3088440 polymorphisms were not found to have any connection with the overall risk of cancer. However, it was observed that the rs3731249 polymorphism exhibited a significant association with the risk of ovarian cancer. Furthermore, the rs11515 polymorphism was found to be significantly associated with the risk of cancer specifically among individuals of Asian descent.^53^

This meta-analysis significantly enhanced the statistical robustness of the findings by aggregating data from multiple studies, and the appropriateness of the included studies adhered to our predetermined inclusion criteria.

Nevertheless, there are specific limitations to consider in this ongoing meta-analysis. Firstly, the outcomes of this meta-analysis were based on unadjusted estimates, whereas a more thorough analysis would require access to individual-level data, allowing adjustments for variables such as age, gender, familial history of malignancy, socioeconomic status, environmental factors, and lifestyle habits. Secondly, the majority of the studies included in this meta-analysis were conducted within the Asian and Caucasian populations. As a result, it was not possible to evaluate the potential impact of the CDKN2A polymorphisms on other populations. Moreover, the subgroup analysis based on ethnicity had small sample sizes for each group, except for Caucasians. Therefore, the determination of the relationship between CDKN2A polymorphisms and the risk of ALL, with respect to ethnicity, remains a subject of ongoing debate. Lastly, the study did not assess genetic and environmental interactions due to insufficient original information from the included studies. Therefore, it is necessary to further validate the conclusions of this study by conducting a large-scale investigation encompassing diverse populations.

In summary, the compiled data shows that the C allele in rs3731217 and the T allele in rs3731249 are associated with protection and susceptibility to ALL, respectively. By identifying the specific genetic variations within the CDKN2A gene in pediatric patients, it becomes possible to tailor treatment plans and accurately predict prognosis. For instance, children who exhibit certain CDKN2A gene polymorphisms may experience favorable outcomes with the early treatment using palbociclib or other pharmaceutical interventions that target CDKN2A gene-related pathways.

## Declarations

### Ethics approval

This article does not contain any studies with human participants or animals performed by any of the authors.

### Consent for publication

NA.

### Availability of data and material

The datasets generated during and/or analyzed during this study are available from the corresponding author on reasonable request.

### Funding

There was no funding source.

### Authors' contributions

Study concept and design: MA, and FA. Data analysis and interpretation: SAD, MB, and MGT. Drafting of the manuscript: RB, AS, and HA. Critical review of the manuscript: HN, AM, and HN. Statistical analysis: KA, SAD, and HN. Administrative and technical support: MA, and SAD. Study supervision: SAD, and FA. All authors read and approved the final manuscript.

## Conflicts of interest

The authors declare that they have no conflict of interest.
